# Functional Foods in Clinical Trials and Future Research Directions

**DOI:** 10.3390/foods14152675

**Published:** 2025-07-29

**Authors:** Zsuzsa Emma Hajzer, Walaa Alibrahem, Nihad Kharrat Helu, Csaba Oláh, József Prokisch

**Affiliations:** 1 Department of Integrative Health Sciences, Faculty of Health Sciences, Institute of Health Sciences, University of Debrecen, Kassai út 26, 4028 Debrecen, Hungary; hajzer.zsuzsa@etk.unideb.hu; 2 Doctoral School of Health Sciences, University of Debrecen, Egyetem Tér 1, 4028 Debrecen, Hungary; walaaeb@mailbox.unideb.hu (W.A.); olahcs@gmail.com (C.O.); 3 Mathias Institute, University of Tokaj, Eötvös Str. 7, 3950 Sárospatak, Hungary; 4 Neurosurgery Department, Borsod County University Teaching Hospital, Szentpéteri Kapu 72-76, 3526 Miskolc, Hungary; 5 Faculty of Agricultural and Food Sciences and Environmental Management, Institute of Animal Science, Biotechnology and Nature Conservation, University of Debrecen, Böszörményi Street 138, 4032 Debrecen, Hungary; jprokisch@agr.unideb.hu

**Keywords:** functional foods, clinical trials, probiotics, prebiotics, postbiotics, omega-3 fatty acids, antioxidants

## Abstract

Clinical trials serve as a cornerstone in the meticulous assessment of the efficacy and myriad health benefits that functional foods offer. These trials are not merely confined to the specific domain of functional foods; rather, they resonate throughout the expansive realms of nutrition science and public health, illuminating the intricate interdependencies that exist among these disciplines. This interconnectedness is becoming increasingly apparent, emphasizing the significant influence of scientific inquiry on fostering healthier dietary habits and shaping well-informed public health strategies. Functional food clinical trials yield essential insights into the potential of functional foods to enhance health outcomes, thereby playing a pivotal role in the prevention of various ailments and substantially elevating the quality of life for individuals in diverse contexts. By delivering consistent and compelling results, these trials bolster the foundational knowledge requisite healthcare practitioners to navigate dietary decisions wisely. Ultimately, the impact of such trials transcends individual health, contributing to the collective well-being of communities. They serve as a vital link between scientific progress and practical implementation, ensuring that the benefits of research are seamlessly integrated into everyday dietary practices, thereby promoting a healthier society at large.

## 1. Introduction to Functional Foods

As we step into the 21st century, the demand for food is rising rapidly throughout the world. In the poorest nations, an insufficient food supply leads to the consequences of food deficiency. In developed countries, overnutrition leads to chronic diseases. Therefore, the concept of food is shifting from the maintenance of health to the promotion of better health [1]. In other words, food products are designed not only to provide energy and nutrients, but also to reduce the risks of some diseases [2]. Functional foods are thus defined as foods with potential health claims [3]. Japan, the United States, and Europe show the highest proportion of functional food consumers [3].

Japan has a long history of using foods with health benefits and Japanese people are well known for their longevity, Major health claims in the new regulation system are associated with blood flow, body temperature, Body Mass Index, eyes, fatigue, joint muscles, memory, stress, and sleep [4]. In some European countries, health claims are used in over 15% of commonly eaten packaged foods [5].

**Figure 1:**
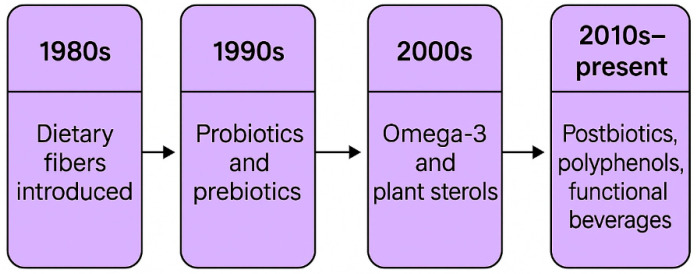
Chronological overview of functional food concept’s evolution.

Functional foods are defined as foods or food components that provide an additional physiological benefit that may reduce the risk of disease or promote health according to the Food and Drug Administration (FDA) [6]. A chronological overview of functional food concepts evolution is shown in Figure 1.

Studies have shown that functional foods have health benefits for chronic disease prevention [7,8], for slowing the aging of organs [9], for maintaining and enhancing aesthetic appearance (such as of the skin, hair, and feet) [10], and for controlling body weight [11]. Moreover, preclinical and clinical studies have shown that intake of functional foods could have an effect on the prevention of chronic diseases, especially cancer, neurological diseases, cardiovascular diseases, and gastrointestinal tract disorders [8].

While several reviews have addressed functional food classifications and health benefits, few have integrated clinical trial design and regulatory perspectives in a single comprehensive synthesis. This review uniquely aims to bridge these domains by highlighting the methodological complexities and limitations in designing and interpreting clinical trials for functional foods, identifying regulatory and market-related gaps that hinder the translational potential of scientifically validated functional foods. By addressing these gaps, this review contributes a multidimensional framework for future research, innovation, and policy in functional food science, facilitating a more effective transition from lab to label and to lifestyle.

## 2. Importance of Clinical Trials

Clinical trials conducted for functional foods share common features, limitations, and challenges with pharmaceutical trials [12]. Both types of trials are implemented to quantify the efficacy and safety of a product for human health, as shown in Table 1.

The evaluation of food products for health-related claims based on health records requires more sophistication than that of pharmaceutical drugs. Functional foods are susceptible to numerous confounding variables and significant difficulties in study designs, which may influence the observed treatment effects [12]. Data reported by the clinical trials conducted for functional foods may be subject to interpretation bias [12]. Studies assessing food components or non-chemical health products for effects on clinical health outcomes are rare, and only a few have generated a level of evidence high enough to reach health claims of significant size in European markets [13]. The possibility of food product studies generating evidence that can be applied to other populations or settings is challenged by several issues that are either inherent properties of foods or study design characteristics associated with food product proposals [14]. The mean treatment effects for most clinical outcomes reported by the food trials are small and typically in the category of no significant effects, with the exception of a few large cohort studies employing innovative designs [13,15].

**Table 1:** Differences between pharmaceutical and functional food clinical trials.

Feature	Pharmaceutical Trials	Functional Food Trials	References
Primary goal	Efficacy and safety	Health promotion and prevention	[16]
Study design complexity	High (controlled, standardized)	High (dietary habits vary)	[15]
Regulatory oversight	Strict (FDA, EMA)	Emerging, diverse globally	[17]
Confounding variables	Minimally present	Highly present (diet, lifestyle)	[17]

## 3. Bioactive Compounds in Functional Foods

Functional foods are broadly defined as foods or food components that provide health benefits beyond basic nutrition [8].

Generally, functional foods are rich in bioactive compounds like omega-3 fatty acids, probiotics, prebiotics, postbiotics, polyphenols, flavonoids, carotenes, lycopene, folic acid, inositol, glycolipid, phytosterols, phosphatidylcholine, oleic acid, and certain antimicrobial peptides [18]. Bioactive compounds are natural or synthetic substances found in foods not ordinarily found in the diet, such as essential nutrients like vitamins or minerals, but which alter metabolic processes and cellular signaling through interaction with enzyme systems or cellular receptors that help promote health or reduce the risk of disease [19].

The consumption of health-promoting functional foods may improve health, longevity, and quality of life, and reduce the incidence of chronic diseases such as cancer, diabetes, coronary heart disease, and chronic obstructive pulmonary disease [20]. An increasing interest has arisen in the chemoprevention and treatment of these diseases by dietary agents, leading to potential opportunities for the development of food-based functional foods with nutraceutical applications. Antioxidant compounds show wide-ranging and potent biological activities, and are considered to be effective agents in the prevention of oxidative-stress-associated diseases (including cancer) [21,22].

Several dietary strategies for the prevention of chronic diseases have been recommended. These preventive strategies can be grouped into (1) health promotion and education and (2) dietary modification by selection of bioactives [23]. Well-designed functional foods with bioactive principles may be a practical and acceptable means to help individuals modify their dietary habits, and can play a vital role in health promotion and disease prevention [24]. Moreover, functional food consumption is recommended in dietary guidelines issued by various countries as advantageous for human health [25].

### 3.1. Probiotics

Probiotics are live microorganisms that confer health benefits on the host when administered in adequate amounts. They are usually dietary supplements or fortified foods containing probiotic bacteria. Strains belonging to the genera Bifidobacterium and Lactobacillus are the most widely used probiotic bacteria [26].

Functional food products containing probiotics have attracted growing interest from consumers and food industries [16]. Generally, probiotics are expected to confer superior health benefits than prebiotics alone; however, the exact mechanisms of action of probiotics’ health benefits require further elucidation [27]. Commercial probiotics differ in terms of provenance, strains, applications/health benefits, formulations, food vehicles, viable cell count, and storage conditions. As with any food ingredient, knowledge on the safety evaluation of probiotics is critical [28]. It is essential to determine both emulsification and incorporation of probiotic strains into the fermented beverage product by secretion and use of casein protein variants, respectively. The use of proteins from bovine milk has been associated with dietary exposure to several bioactive peptides [29]. Enterprises involved in the production and marketing of probiotic products are recommended to comply with the guidelines on manufacturing, quality control, labeling, testing, and advertising of probiotic foods. A missing safety assessment scheme for probiotics marketed as Ingredients Generally Added (IGAs) is required [30].

Several probiotic preparations have been evaluated in adults and children, demonstrating some benefits for symptoms associated with gastrointestinal (GI) disorders. Dissolving one probiotic capsule in an artificial gastric acid increased GI transit time, indicating that probiotic enteric capsules could protect probiotics against gastric acid [31]. Transglutaminase-based capsules effectively encapsulated probiotics and preserved the viability of these probiotics under simulated GI conditions. Another study suggested that probiotics could reduce pro-inflammatory cytokines such as IL-6, IL-8, and tumor necrosis factor- alpha (TNF- α) while upregulating anti-inflammatory cytokines such as IL-10, attenuating mucosal damage and improving gut microbiota. It was emphasized that more clinical studies are required to confirm the probiotic efficacy, pharmacokinetics, and identification of bioactive compounds [32].

In recent years, there have been ups and downs in the commercial recommendations for probiotics in functional gastrointestinal disorders (GI functional disorders) [31]. 31Evaluating probiotic efficacy was complex because multiple probiotic strains and different doses of probiotics could be administered as adjunctive therapies. Currently, baby- and child–adult-specific probiotics have been more popular due to the better microbiota modulation effects and targeted clinical trials targeting screening for probiotics to evaluate the cumulative history of safe consumption [33].

### 3.2. Prebiotics

Prebiotics are a carbohydrate-based substance that is not digested in the upper gastrointestinal tract but is fermented and selectively utilized by bacteria in the colon [34]. Prebiotics, like inulin, affect gut microbiota such as Bifidobacterium adolescentis and Faecalibacterium prausnitzii. Baseline microbiota activity and initial bifidobacteria counts influenced responses to prebiotic dosing in healthy subjects using diet recommendations and 2, 6, or 10 g of inulin [35,36]. A reduced-fat yogurt provides low doses of prebiotics to study gut microbiota but not within dietary recommendations. There are still fewer studies on a complete mixed meal containing commercially available sources of prebiotics and resistant starch [37]. Brands can identify dietary patterns of prebiotics that matter most to gut microbiota [38]. Fermented dairy products have numerous health benefits, such as better balance of colonic microflora, bowel habits, and treatment of diarrhea [39]. Clinical trials on fermented milk products containing probiotics and prebiotics have limited gastrointestinal health effects [40]. Hence, a study design is proposed for symbiotic fermented milk product development containing Lactic Acid Bacteria (LAB) and an inulin-based prebiotic as ingredients [41].

### 3.3. Postbiotics

Postbiotics have recently garnered significant interest in a multitude of clinical trials owing to their numerous potential health benefits and diverse applications [42]. Scientists are increasingly allocating their resources and expertise to understand how these powerful and beneficial compounds can lead to the effective and sustainable maintenance of a healthy and balanced microbiome [43]. With ongoing and extensive studies, their prospective applications in various therapeutic strategies are becoming clearer, more compelling, and increasingly vital in the quest for optimal health. The research landscape surrounding postbiotics is expanding rapidly, revealing even more potential mechanisms by which they can improve our health and support various bodily functions [44].

### 3.4. Omega-3 Fatty Acids

The brain is highly enriched with long-chain omega-3 fatty acids, phosphatidylserine and sphingomyelin. There is a growing trend in older people to use supplements to boost cognitive health [45]. There are still some conflicting results regarding its protective effects against age-associated memory decline, cognitive impairment and Alzheimer’s disease risk, as some trials failed to show beneficial effects [46]. Such conflicting results could be due to differences in study populations, doses applied, and the types of long-chain omega-3 fatty acids used in those studies [47]. Clinical intervention trials were conducted in cognitively healthy older people to test the hypothesis that dietary supply of 1720 mg/d long-chain omega-3 fatty acids can ameliorate working memory [48,49]. There are several ongoing clinical trials within the population of healthy and cognitively healthy middle-age to older adults testing whether daily supplementation for periods from 5 to 90 weeks can improve some aspects of mood, executive functions, and cognition. Moreover, supplementation did not alter insights into cognitive changes for most participants. Nonetheless, clinical morbidity was found to exert either beneficial or deleterious effects on cognition, rendering certain individuals more at risk for cognitive changes and deteriorations [49].

The health benefits of long-chain n-3 polyunsaturated fatty acids (n-3 LC PUFA) consumption have been reported in both experimental animal studies and human studies for conditions such as CVD, ARDS, rheumatoid arthritis, diabetes mellitus, and dysmenorrhea. Fish, particularly oily fish, are the most abundant and well-recognized source of food-derived n-3 LC PUFA, but n-3 LC PUFAs are also present in some seafood and seaweed [50]. The CVD health benefits of fish consumption have been substantiated to the point that the US Food and Drug Administration approved a qualified health claim for n-3 LC PUFA dietary supplements and coronary heart disease, which has not occurred for any other ingredient. Omega-3-enhanced foods are also increasingly popular, but few human studies have examined their impact on measures of n-3 PUFA status and cardiovascular risk factors, and no studies have been conducted in a naturalistic setting [51]. In the GISSI-Prevenzione study, the effects of acute post-myocardial infraction (post-MI) supplementation with n-3 LC PUFA for three years were evaluated. Relative to control group participants, intervention group participants were 61% less likely to have died of any cause, and significantly less likely to have died from CVD, died suddenly, had an MI, or had an event of any other type. The participants in the treatment group were less likely to be taking cardioprotective medications and had a more severe CVD risk profile within the cohort, yet were 73% less likely to have had an MI [52]. It is unclear from this study how many deaths in control participants were attributable to CVD, but the yearly rate was 35, and thus many lives could be saved by n-3 LC PUFA supplementation. Omega-3 PUFAs may also improve other risk factors, such as reducing blood pressure, hemoconcentration, platelet adhesion, and thromboxane production. The direct effects on the heart may include increased endothelial vasodilator function, arterial compliance, and heart rate variability [53].

### 3.5. Antioxidants

Antioxidants are estimated to be about 20,000 kinds of chemical molecules and about 150 kinds are well characterized and widely studied as functional foods [54]. On the other hand, health claims on anti-oxidative stress by taking these antioxidants are now promoted, while not enough scientific evidence supports the claim on health improvement and disease prevention by them [55]. It is of the utmost urgency to evaluate the medical, health preventive and health promotional effects of functional foods for the society with aging problems and the government with reimbursement functions [56]. It has been reconfirmed that oxidative damage occurs induced by the administration of pro-oxidants and preventing oxidative damage can result in improved health and prevention of diseases in clinical trials using such substances [57]. With respect to reactive oxygen species (ROS), they are directly or indirectly related with oxidative damage of nucleic acid, lipids, amino acids, proteins and metals, and can cause various diseases [58].

### 3.6. Peptides

Peptides refer to short amino acid sequences, which serve as building blocks of proteins but are themselves bioactive molecules with myriad roles in physiology. Peptides are recognized for their ability to serve as blood pressure regulators primarily because they possess angiotensin-converting enzyme (ACE) inhibitory activity [59]. Bioactive peptides are synthesized during fermentation or through enzymic hydrolysis of dietary proteins, releasing sequences with discrete biological functions in the human body [60]. Incorporation in functional beverages, e.g., probiotic-fortified beverages, of casein protein variants and bovine milk proteins has been attributed to dietary consumption of such peptides with potential effects on cardiovascular disease and intestinal integrity [61]. Peptides are also implicated in emulsification processes and insofar as they contribute to the incorporation of probiotics into food matrices, thereby improving product stability and bioavailability. The modulatory functions of peptides in the metabolic and cellular signal-transducing pathways render them significant entities in the prevention and maintenance therapy for chronic disease [59].

### 3.7. Phenolic Compounds

Phenolic compounds, including flavonoids and phenolic acids, are bioactive phytochemicals prevalent in tea, berries, cocoa, and olive oil. They are known for their potent antioxidant and anti-inflammatory activity and contribute to chronic disease prevention, including cardiovascular diseases and certain cancers, through modulation of cellular signal pathways and reactive oxygen species scavenging [62]. While preclinical results are promising, it remains challenging to translate these actions into robust clinical endpoints due to low bioavailability, dietary matrix effects, and inter-individual metabolism [63]. Micro- and nano-encapsulation technologies have recently emerged as hopeful methods for stabilizing and optimizing the delivery and efficacy of phenolic compounds in functional foods. For this reason, phenolics are central to functional foods R&D and dietary interventions aimed at improving public health [64].

### 3.8. Glucosinolates

Glucosinolates are sulfur-bearing phytochemicals in cruciferous foods such as broccoli, cabbage, and Brussels sprouts [65]. Primary glucosinolate health effects come as enzymatic breakdown products, particularly sulforaphane, which has potent detoxifying, anti-inflammatory, and anticancer properties. Sulforaphane triggers phase II detoxification enzymes and regulates redox-sensitive transcription factors such as the Nrf2 and adds towards cellular defense and cancer chemoprevention [66].

Despite vigorous preclinical evidence, translation of glucosinolate efficacy into human health impacts has proven complex. Factors including thermal inactivation of plant myrosinase during heating, inter-individual differences in gut microbiota, and divergent metabolism significantly impact bioactivation and bioavailability [67]. Microencapsulation and delivery system technologies are promising for stabilizing sulforaphane as well as optimizing site-specific delivery in functional food systems [68].

With their chemopreventive potential as dietary agents, glucosinolates and their degradation products continue as focal points in functional foods research but they should be subjected to vigorous clinical trials before establishing their therapeutic potential in human beings [67].

### 3.9. Carotenoids

Carotenoids, particularly β-carotene, lutein, and lycopene, are ubiquitous plant pigments responsible for the vivid coloring in fruits and vegetables such as carrots, tomato, and leafy greens. Besides cosmetic relevance, they are recognized for their significant health effects [69]. Functioning as very potent antioxidants, carotenoids inactivate reactive oxygen species (ROS) and therefore neutralize oxidative harm to cellular macromolecules including lipids, proteins, and DNA. Due to this antioxidant action, they are credited with the reduction in the incidence of chronic diseases such as age-related macular degeneration, cardiovascular disease, and certain cancers [70].

Carotenoids are also involved in immune function and serve as significant bioactive molecules in the preventive nutrition and functional foods context [71]. However, their health implications are closely related to their bioavailability, this being influenced by numerous factors such as foods’ matrix, dietary oil availability, and processing. Heat processing and co-consumption with oils, for instance, will elevate carotenoid breakdown and micellar absorption, but fiber content and oxygen exposure can reduce their stability and bioaccessibility [72].

### 3.10. Phytosterols

Phytosterols are plant bioactive compounds present naturally in plant foods and are used widely in functional foods due to their demonstrated efficacy in decreasing low-density lipoprotein cholesterol (LDL-C) [73]. Structurally similar to cholesterol, they decrease intestinal absorption of dietary cholesterol and therefore cause clinically significant reductions in serum LDL-C at a daily dose of 2 g. For this reason, cholesterol-lowering health claims have been authorized by regulatory agencies such as the FDA and EFSA for foods fortified with phytosterols [74].

These sterols are incorporated in several functional foods like fortified margarines, milk products, and beverages in the form of preventive nutrition and cardiovascular disease risk management. But their therapeutic efficacy must be ensured through regular and consistent daily consumption, and there may be differences in response in individuals due to genetic and nutritional factors [75].

### 3.11. Alkaloids

Alkaloids are a structurally diverse category that primarily contains nitrogen and are intrinsic to many plant-derived dietary items [7]. For example, theobromine in cocoa and caffeine in coffee are compounds that demonstrate established physiological impacts on the central nervous system, activating and affecting heart rate and energy metabolism. In addition, these compounds establish their role as functional ingredients [8]. Among them, caffeine is one of the most common alkaloids, which enhances mental vigilance and focus through inhibition of adenosine-mediated neuronal suppression. Moreover, it can increase thermogenesis and lipid catabolism, making it a regular ingredient in fat-loss dietary products [76].

In the field of functional food development, alkaloids present the benefit of producing fast and measurable biological responses, thus increasing market attractiveness and product uniqueness [77]. However, their tight dose–response window demands controlled composition strategies [8]. Indeed, overconsumption leads to harmful physiological consequences such as insomnia, tachycardia, and anxiety, particularly among vulnerable populations or patients with known cardiac disorders [76].

Overall, alkaloids are recognized as effective ingredients in functional nutrition that link cognitive stimulation with metabolic regulation, emphasizing the importance of data-driven product design and moderated intake within wellness-focused dietary solutions [77].

### 3.12. Saponins

Saponins are a multifaceted group of compounds characterized by their amphiphilic glycoside nature, commonly occurring in plant-derived dietary sources such as legumes, quinoa, and ginseng. They are noted for their surface-active properties that allow for stable foam formation in water-based environments and exhibit multiple biofunctional properties of interest in the field of nutraceutical and functional food development. Among their well-documented biological effects is the reduction in serum cholesterol levels, which is attributed to their affinity for bile acid sequestration in the gastrointestinal tract, thereby enhancing fecal elimination of bile acids and stimulating hepatic biosynthesis of bile acids from cholesterol, ultimately lowering circulating cholesterol concentrations [78,79].

In addition to their effects on lipid metabolism, saponins demonstrate immune-modulating and inflammation-suppressing activities, positioning them as potential agents in prophylactic dietary strategies. These effects are facilitated by the upregulation of cytokine synthesis and the activation of phagocytic immune cells, thus enhancing innate immunity. Moreover, saponins exhibit chemopreventive and anticancer properties via pathways that regulate cell proliferation and promote programmed cell death in various cancer cell lines [80].

However, the integration of saponin-containing compounds into functional food formulations is limited by their inherent bitterness, as well as the possible disruption of lipophilic nutrient bioavailability. Furthermore, concerns regarding consistency in composition and biological availability continue to be focal points in the advancement of nutraceutical science [80]. A comprehensive overview of key bioactive compounds is presented in Table 2.

## 4. Regulatory Framework

The remarkable health benefits of functional foods are well established, although they have, until recently, been considered “non-drug” or not potent enough to produce positive clinical responses. Historically regarded as a luxury, they are now considered ready-to-use. With reasonable efforts, some functional foods can bring clinician-assessed clinical benefits, mirroring the performance of chemical drugs [4]. However, strict guidelines for clinical evaluations of functional foods need to be established.

In Japan, functional foods are managed under two official systems. The Foods for Specified Health Uses (FOSHU) program, introduced in 1991 by the Ministry of Health, Labour and Welfare, requires manufacturers to submit scientific evidence on both safety and efficacy. Only after rigorous assessment can a product display approved health claims. In April 2015, Japan also launched the Foods with Function Claims (FFC) framework, which simplifies market entry; it allows businesses to self-submit substantiation, via clinical trials or systematic reviews, and notify the Consumer Affairs Agency, without awaiting individual pre-approval [4,48,81].

In China, functional foods are categorized as “Health Foods” and overseen by the National Medical Products Administration (NMPA). To market such foods, the producers must undergo either a registration route, required for novel ingredients or imports, or a notification system for existing, approved ingredients. The product’s claimed health function must adhere to a government-approved directory of roughly two dozen functions [82].

Within the European Union, the use of health-related claims on functional food products is tightly controlled under Regulation (EC) No. 1924/2006, effective since July 2007. All claims must undergo scientific validation and gain the approval of the European Food Safety Authority (EFSA). The regulation aims to harmonize standards across member states, safeguard consumers against misleading marketing, and maintain a transparent EU register of permitted claims [83,84].

**Figure 2:**
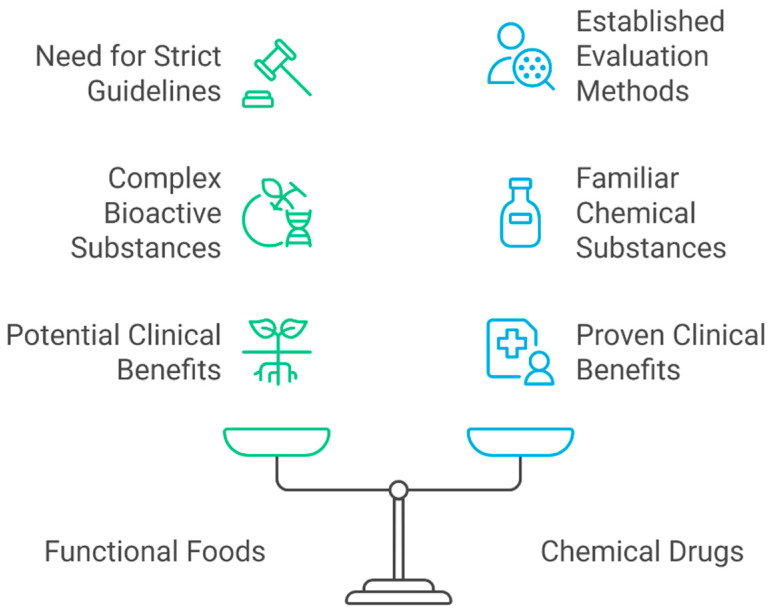
Balancing functional food and chemical drugs.

Unlike chemical drug substances, which are generally considered “familiar”, functional foods consist of several bioactive substances with varied degrees of affluence and combinations, as shown in Figure 2. Compound functional foods comprising multiple ingredients might have a potential for unexpected reactions [85]. In other cases, how to consider the use of foods with evaluated bioactive functional substances as industrially important functional foods must be studied. The oversupply of functional foods with little clinical investigation is another concern. The question of what evidence could be accepted, as assurance of a functional food, must be raised [86]. These issues require a regulatory existence for clinical evaluations of functional foods. However, functional foods can be observed in different states in different countries. If solid grounds are taken as industrial opportunities, inevitable confusion and concern will arise. A fine categorization of deliberations for functional foods in several countries might provide a good clue [4].

## 5. Functional Foods in Clinical Trials

Overall, clinical trials are designed to answer a scientific question using hypotheses. Various forms of research questions exist. Early-phase trials primarily aim to test a new drug in a small number of selected patients to learn about its efficacy, dosing, toxicity, and safety (Phase 1 trials), whereas later-phase trials usually involve a larger patient population predetermined by the power calculation (Phase 2, 3, or 4 trials) [87]. In addition, there are different trial designs to answer different concepts and hypotheses, as shown in Table 3.

The study population should be predetermined according to relevant factors with respect to the trial’s objectives. Healthy individuals or patients with specific disorders who are willing to consume the test product should be included. In addition, inclusion and exclusion criteria to avoid confounding cases should also be carefully defined [88].

These steps, ranging from objective formulation to participant selection and baseline assessment, are part of a structured clinical trial timeline typically followed in functional food evaluation, as shown in Figure 2. In trials for dietary treatments, these criteria should be complemented with relevant dietary factors such as habitual diet, diet-related habits and practices. For example, in trials for functional foods or nutraceuticals, subjects with food allergies, concurrent consumption of other dietary supplements, history of following dietary regimens or restrictions should also be excluded [89]. Dietary intake data should be quantitatively assessed, with prescribed weight and portion sizes of solid food as well as specific methods and times of food preparation needing to be documented to maintain supervised consumption of an impact control [90].

Sufficient recruitment of participants within the desired inclusion/exclusion criteria is essential for trial validity, generalizability and meeting regulatory requirements. Recruitment could take the form of active approaches, i.e., advertisements in health food magazines or producing posters for centers with a known vested interest in functional foods, or passive approaches such as advertisements in newspapers. All researchers should be trained to verify access to a specified recruitment pool or envelope [91]. Unsuitable volunteers could be categorized as temporary or permanent. When the participants are detected as unsuitable for screening, alternatives could be continued with until the required quota is reached [92]. The recruitment of participants is crucial in terms of trial validity, representativeness, generalizability and to meet regulatory requirements [93].

Systematic random selection of individuals attending or frequenting the venues considered for a trial has been shown to be effective at identifying initial contact, opting eligibility and permitting easier participant recruitment at otherwise inaccessible locations [94]. Published protocols and experiences from regulators and within the field regarding consumer recruitment may also be informative for food scientists undergoing similar trials [95].

With the increasing number of functional food products in the marketplace, clinical trials aimed at testing these products are rapidly increasing. The objective of these trials is to afford product effectiveness statistical justification. In the past, clinical trials on functional foods have been conducted, occasionally producing interesting results [96]. However, a stringent statistical inferential methodology to ensure product effectiveness has so far not been pursued [97]. As a consequence, there are currently a large number of positive findings from functional food product tests in the marketplace. However, it can be argued that it is possible to find positive results by simply ensuring an adequate design of the test, independently of the product itself [98]. Therefore, much effort is currently dedicated to inferential statistics, with emphasis on finding a procedure for testing product effectiveness that guarantees low levels of false positive errors and high levels of positive product testing as a function of the effect size [99]. Several clinical trials demonstrating effects of functional foods on human health are summarized in Table 4.

**Table 3:** Summary of clinical trial designs for evaluating functional foods.

Trial Design	Primary Objective	Advantages	Limitations	References
Randomized controlled trial (RCT)	Assess efficacy and safety under controlled conditions	High internal validity, minimizes bias, provides high-quality evidence, gold standard in clinical evaluation	Time-consuming, resource-intensive, limited for pre/post-market evaluations, consumer behavior variability introduces bias	[100,101,102]
Crossover trial	Compare treatments within the same subjects	Requires fewer participants, reduces inter-subject variability	Risk of carry-over effects, longer study duration	[103,104]
Parallel-group trial	Compare outcomes between separate groups	Simple design, no carry-over effects	Requires larger sample sizes	[105,106]
Open-label trial	Evaluate treatment effect when blinding is not feasible	Easier to conduct, reflects real-world conditions	Increased risk of observer and participant bias	[107,108]
Blinded (single/double/triple)	Reduce bias in reporting and assessment	Enhances credibility of findings	Complex logistics, not always feasible in nutrition studies	[109]
Observational cohort study	Monitor outcomes in natural settings over time	Reflects real-life conditions, useful for long-term effects, explores prevention guidelines	Cannot establish causality, more confounding factors	[110,111]
Cross-sectional study	Examine correlations at a single point in time	Useful for identifying associations and informing hypotheses	Cannot establish causality, depends on timing of data collection	[112,113]

**Table 4:** Effects of functional foods on human health in several clinical trials.

Authors (Year)	Design (N, Duration)	Key Findings	References
Timmers et al. (2016)	Double-blind, placebo-controlled crossover RCT; 17 T2D patients; 30 days (150 mg/d resveratrol vs. placebo)	Resveratrol did not improve hepatic or peripheral insulin sensitivity. It did enhance mitochondrial function but had no effect on insulin resistance.	[114]
Zare et al. (2019)	Triple-blind RCT (parallel groups); 140 T2D patients (BMI stratified); 3 months (500 mg × 2 daily cinnamon vs. placebo)	Cinnamon supplementation improved BMI and body fat, and significantly reduced fasting/postprandial glucose, HbA1c, fasting insulin and HOMA-IR. Total cholesterol, LDL and HDL cholesterol also improved (triglycerides unchanged). Benefits were greater in patients with BMI ≥ 27.	[115]
Dehghan et al. (2013)	RCT, placebo-controlled; 49 women with T2D; 8 weeks (10 g/d inulin vs. maltodextrin)	High-performance inulin significantly lowered fasting glucose, HbA1c, total cholesterol, triglycerides, LDL and LDL/HDL and TC/HDL ratios, while raising HDL. No significant changes in the placebo group.	[116]
Wolever et al. (2021)	Double-blind RCT; 207 adults (LDL 3.0–5.0 mmol/L); 4 weeks (3 × 1 g high-MW oat β-glucan drink/day vs. rice powder)	Oat β-glucan lowered LDL-C and total cholesterol. It also reduced non-HDL and TC:HDL ratio, translating to ~8% reduction in 10-year Framingham CVD risk. No changes in HDL, TG, glucose or insulin.	[117]
Ried et al. (2016)	Double-blind RCT; 88 patients with uncontrolled hypertension; 12 weeks (aged garlic extract 1.2 g/d vs. placebo)	Aged garlic extract significantly reduced blood pressure. Trends suggested improvements in central hemodynamics, arterial stiffness and inflammatory markers, though most were non-significant.	[118]
Atefi et al. (2018)	RCT; 77 women with T2D; 8 weeks (30 g/day: olive oil vs. canola oil vs. sunflower oil)	Replacing saturated-fat oil with canola or olive oil lowered inflammation. Both canola and olive oil groups showed significant reductions in CRP versus baseline and vs. sunflower oil (*p* < 0.05). No significant differences were seen in blood glucose or other lipids between groups.	[119]
Stote et al. (2020)	Double-blind RCT; 52 men with T2D; 8 weeks (22 g freeze-dried blueberry powder/day vs. placebo)	Blueberry intake improved glycemic and lipid markers. HbA1c was significantly lower in the blueberry group vs. placebo. Fructosamine, serum triglycerides, and liver enzymes (AST, ALT) also fell significantly with blueberries. Fasting glucose, insulin, cholesterol and CRP were unchanged.	[120]
Richter et al. (2021)	Double-blind crossover RCT; 40 overweight/obese adults with elevated BP; 8 weeks cranberry juice (500 mL/d) vs. placebo, 8-week washout	Cranberry supplementation had modest cardiovascular benefits. It did not change central BP but lowered 24 h ambulatory diastolic BP during daytime. It altered lipoproteins: large LDL particles and LDL size both increased with cranberry vs. placebo. No effect on central SBP or LDL-C concentration.	[121]
Chuengsamarn et al. (2012)	Double-blind RCT; 240 prediabetic adults; 9 months (750 mg curcumin/day vs. placebo)	Curcumin prevented progression to T2D *p* < 0. Curcumin also improved β-cell function (HOMA-β ↑, HOMA-IR ↓) and raised adiponectin. FPG and 2h-glucose remained stable in curcumin group but rose in controls.	[122]
Chatree et al. (2021)	RCT; 40 obese adults; 8 weeks (300 mg EGCG/day vs. placebo)	EGCG (green tea extract) significantly lowered metabolic risk factors. After 8 weeks, fasting triglycerides, systolic BP and diastolic BP. No significant changes in body weight, glucose or insulin were observed.	[123]
Berryman et al. (2015)	Controlled-feeding crossover RCT (6 wk each); 48 adults with high LDL-C; 1.5 oz/d almonds vs. isocaloric muffin	Almonds markedly improved lipids. Almonds also reduced abdominal fat, despite no change in body weight.	[124]

## 6. Challenges in Conducting Trials

Food-based interventions are complex because they may vary widely in composition, sources, forms, preservation, preparation, and processing. Thus, careful documentation is necessary but very difficult and challenging to achieve [125].

Also, the nature of an active food and the confounding variables and complexity that it brings represent challenges for the ascertainment and standardization of the mode of action of foods in the absorption, distribution, metabolization and excretion of food-derived functional compounds [126]. For example, considerable evidence exists on the health consequences of food effectiveness depending on its matrix. Others show great variability for, e.g., cereal bran that includes both soluble and insoluble dietary fibers, which may impact the effectiveness of cereal consumption. Alternatively, soya isoflavones might act in a different food format as compared to a supplement format [127].

In addition to these complexities, there is a fundamental limitation related to the time that counselling agents or professionals dedicate daily to dietary guidance in comparison to the time individuals spend ingesting foods [128].

### 6.1. Participant Compliance

The evaluation of functional foods via clinical trials poses many challenges. Due consideration must also be given to participant compliance with adherence monitoring and participant retention strategies, which are both critical factors in trial success [129]. Investigators must ensure that potential participants have sufficient interest to modify habitual consumption patterns as part of trial entry considerations. The willingness of participants to receive food products via home delivery should also be confirmed to mitigate potential issues with low compliance [130]. As adjustments to the provided intervention may not be practical or feasible at a later stage in the dietary intervention study design, recruitment considerations must critically include food product palatability. To fully evaluate food products for palatability, screening by trained taste panelists and subsequent participant free-choice taste preference and pair preference testing via acceptability trials can be implemented before commencing trials [131]. When creative testing strategies are employed, participant preferences can be incorporated into intervention design, helping to mitigate a major cause of trial failure. Creating simple systems for anonymity and methods for training participants for blinding testing would enhance the quality of data collected. Specifying food products, test preparation, delivery and timing, participant criteria, and screening for excitement about the product type and cultural acceptance in these decisions would augment co-design [132].

### 6.2. Funding Issues

As a consequence of the previous issues encountered in funding clinical trials for functional foods, only a few European companies are currently developing functional foods and, indeed, have a reduced pipeline of novel products [133]. One of the major barriers to efficient clinical development is the market exclusivity period, which, in the EU, is only 5 years for functional foods. In other regions, a shorter or no exclusivity period is granted. Since the costs of registering a new functional food can be very high, projected yearly revenues should be sufficiently higher than these costs to warrant investment in clinical testing [134]. Thus, unless an application for patent extension can be made on the basis of new knowledge generated in clinical studies, a health claim on a novel functional food will have a very short period of exclusivity, resulting in only small revenues and ruling out financial viability for developing companies to invest in large RCTs. In addition, considerable resources are needed to prepare regulatory submissions, particularly for health claims, where dossiers can easily consist of thousands of pages [130]. Unlike pharmaceuticals, there is no equivalent of a ‘fast track’ regulatory approval process, which would allow companies to submit health claim dossiers in stages. Furthermore, since pharmaceutical companies are generally much larger than food companies, this increases their negotiating power with those responsible for the scientific evaluation of data packages. Moreover, the food industry traditionally works with much lower margins, leading to lower expenditure on R&D [132]. That said, certain food companies have invested considerably more than on clinical development of a single product. However, obtaining a health claim has proven to be a lengthy process, especially for multi-strain probiotics, where several years of neglect of applications have created a backlog. In addition, a ‘non-licensed’ probiotic sector functioning in the grey zone is growing increasingly in Europe. Recurring European regulatory hurdles and off-target processing errors can delay product approvals for years [135].

## 7. Future Directions in Research

Functional foods empower frequently biased choices with respect to the consideration of scientific evidence and understanding of the relationship between food, health, and disease. Functional foods are sophisticated biocomplex matrices that differ from conventional foods due to their chemical composition or format and yield a biological or health benefit [136]. The benefits of functional foods include quality and safety concerns, health claims regulatory framework, scientific evidence of efficacy, controlling information and health claims, as well as the mechanisms of action behind the efficacy of functional food compounds [137]. Functional food research topics should be aligned with emerging health-oriented trends in modern nutrition. Given the issues populations face, health strategies that are based on realistic approaches to dietary modification should be considered, such as areas that focus on foods commonly consumed by populations or development of food technologies to lessen adverse effects of unwholesome foods [138]. Health benefits and mechanism-of-action studies that are tailored to these gaps should be prioritized. There is a need for genome-wide association studies (GWAS) to assess legacy diets within populations or the effects of microbial communities. Further, innovative experimental approaches, such as the use of multi-omics techniques, system biology methods, meta-genomic sequencing, and mapping, should be employed to obtain a holistic view of food–gut–microbiome–host interactions [139]. Furthermore, emerging technologies should be adapted for evermore sensitive detection of food ingredients and metabolites in all matrices and thus, test for effects on their absorption and metabolism. Systematic biobanking strategies and relevant metadata should be developed for archiving and retrieving collective know-how on food effects across populations [138].

The dietary response to some very important diet or nutrient composition measures seems to vary greatly. Some explainable or unexplainable biological mechanisms that could potentially characterize an inter-individual response to a nutrient are germline genetics, transcriptomics, metabolomics, and gut microbiome composition and functionality. Experts thus recommend incorporating more personal data on other levels of ‘omes’ [139].

## 8. Conclusions

Functional foods offer an alternative approach to medicine by providing diets or dietary supplements to minimize the risk of chronic diseases. They are designed to help people seek out daily preventive means rather than waiting until diseases manifest for cures with drugs. While drugs and pharmaceuticals are typically used to cure diseases, they are generally toxic and have side effects. Foods, on the other hand, are normally nontoxic and generally safe for daily consumption, though many people do not eat them for health promotion. Functional foods, which are defined as foods supplemented with functional ingredients that may prevent human diseases and promote health, are either manufactured or natural diets containing functional ingredients. The former are generally manufactured for health prevention, while the latter are whole foods inherently containing functional ingredients. Functional foods may prevent chronic diseases and aging, improve appearance, control body weight, and regulate physiology and biochemistry.

With growing awareness of and desire for the preventive possibility of functional diets, commercial interests in producing functional foods have increased exponentially, with many already on the markets. To help safeguard consumers and to protect potential markets, the food industries in America and Europe have urged government agencies to establish definitions for functional foods, and workshops on defining functional foods have convened in both continents. The European Commission has already released several documents discussing the need for definitions of functional foods. At the same time, the food industries in Asia Pacific countries are highly eager to establish definitions for functional foods, which may help make millions of profits but also safeguard consumers. As far as Asia Pacific countries are concerned, the Eastern view of functional foods and their distribution is quite different from those of the West.

## Figures and Tables

**Table 2:** Summary of bioactive compounds, food sources and health benefits.

Bioactive Compound	Examples	Food Sources	Health Benefit	References
Probiotics	*Lactobacillus*, *Bifidobacterium*, *Saccharomyces*, *Enterococcus*	Yogurt, kefir, fermented dairy products, freeze-dried fruit juice gummy supplements, probiotic powder.	Improves gut health, supports immunity, delivers bioactive peptides, strain-specific protection against enteric pathogens.	[26,27,28,30]
Prebiotics	Inulin, Fructooligosaccharides (FOS), Galacto-oligosaccharides (GOS), Xylo-oligosaccharides	Bananas, garlic, chicory, soy milk, fermented dairy products.	Stimulates growth of beneficial gut bacteria, increases Short-chain fatty acids (SCFAs), reduces harmful bacteria, lowers inflammation.	[36,37,38]
Postbiotics	Short-chain fatty acids (e.g., butyrate), bacterial metabolites	Fermented foods.	Modulates immunity, maintains gut barrier integrity, promotes digestive health, balances microbiome.	[42,43,44]
Omega-3 Fatty Acids	Eicosapentaenoic acid (EPA), Docosahexaenoic acid (DHA), DPA	Fish oil, flaxseed, seaweed oil, marine concentrate.	Supports cardiovascular and cognitive health, improves memory, lowers agitation.	[45,46,48]
Antioxidants	Vitamin C, Vitamin E, Polyphenols, Flavonoids, Melatonin	Berries, citrus, tea, green tea, lycopene.	Reduces oxidative stress, lowers inflammation, prevents oxidative damage to DNA, proteins and lipids.	[55,56,57]
Peptides	Bioactive milk peptides	Fermented dairy products.	Blood pressure regulation (ACE inhibitors).	[59,60,61]
Phenolic Compounds	Flavonoids, phenolic acids	Tea, berries, olive oil, cocoa.	Antioxidant, anti-inflammatory, vascular health.	[62,63,64]
Glucosinolates	Sulforaphane	Cruciferous vegetables.	Detoxification, anti-cancer properties.	[66,67,68]
Carotenoids	Beta-carotene, lutein, lycopene	Carrots, tomatoes, spinach.	Eye health, antioxidant, cancer prevention.	[69,70,71]
Phytosterols	β-sitosterol	Fortified margarines.	Lower cholesterol, clinically significant reductions in serum LDL-C.	[73,74,75]
Alkaloids	Caffeine, theobromine	Coffee, cocoa.	Mental vigilance and focus, thermogenesis, lipid catabolism.	[76,77]
Saponins	Ginsenosides	Ginseng, legumes.	Immune modulation, anti-inflammatory activities, cholesterol-lowering.	[78,79,80]

## Data Availability

No new data were created or analyzed in this study. Data sharing is not applicable to this article.
